# Efficacy of serratiopeptidase after impacted third molar surgery: a randomized controlled clinical trial

**DOI:** 10.1186/s12903-021-01451-0

**Published:** 2021-03-02

**Authors:** Zaid Tamimi, Rola Al Habashneh, Islam Hamad, Mutasim al-Ghazawi, Ala’ Abu Roqa’a, Hamza Kharashgeh

**Affiliations:** 1 Department Oral Surgery and Oral Medicine, Jordan University of Science and Technology, Irbid, Jordan; 2 Department Periodontology, Jordan University of Science and Technology, Irbid, Jordan; 3 Department of Pharmacy, American University of Madaba, Amman, Jordan; 4 Department of Biopharmaceutics and Clinical Pharmacy, The University of Jordan, Amman, Jordan; 5 The First for Research and Development LLC, Amman, Jordan

**Keywords:** Third, Molar, Surgery, Inflammation, Trismus, Swelling

## Abstract

**Background:**

Serratiopeptidase has been clinically used in controlling surgical and non-surgical inflammatory conditions. This study was conducted to assess the therapeutic effect of Serratiopeptidase in patients undergoing surgical removal of impacted mandibular third molar.

**Methods:**

This randomized clinical trial investigated the efficacy of Serratiopeptidase and Paracetamol after surgical removal of impacted third molar for 5 days (n = 67) as compared with an equivalent dose of placebo and Paracetamol (n = 66). Outcome measures were reported pain, trismus and swelling using Laskin method. All outcome measures were recorded on days 0, 1, 2, 4, and 5 post-surgeries.

**Results:**

In this clinical trail 133 patients (mean age 23 years, 54% female) completed the study. Baseline characteristics were comparable across treatment groups. Serratiopeptidase significantly improved trismus compared with control on the 4^th^ day (27.30 ± 7.3 mm and 32.06 ± 7.7 mm, respectively (P < 0.001) Swelling markedly improved, The distance from the lower edge of the earlobe to the midpoint of the symphysis for cases vs control were 111.49 ± 8.1 mm and 115.39 ± 9.9 mm, respectively (P < 0.001). Reported pain, showed no statistical significance difference.

**Conclusion:**

Serratiopeptidase resulted in better inflammation improvement than placebo over 5 days. Further studies are warranted to assess longer-term and clinical outcomes, as well as safety.

**Clinical relevance:**

Serratiopeptidase administered postoperatively helps in improving trismus and swelling after removal of impacted lower third molars.

*Trial registration* The study was registered in ClinicalTrial.gov under the number NCT02493179**.** Registered 1st of June 2015, https://clinicaltrials.gov/ct2/results?cond=serratiopeptidase.

## Background

Impacted third molars are frequently encountered in clinical work. Postoperative sequelae associated with such procedures include pain, trismus and swelling which occur due to the local inflammatory reaction [1]. Proper surgical technique and gentle handling of the tissues will help minimize postoperative inflammation but it will not prevent it [2]. In recent years, several drugs such as corticosteroids and Nonsteroidal anti-inflammatory drugs (NSAIDs) have been prescribed to suppress inflammation [3–5]. However, the use of NSAIDs has been associated with some adverse effects such as gastrointestinal bleeding, renal function disturbance, a reduction in platelet function, shortness of breath, and profound hypotension [6, 7].

Alternative NSAID formulations like celecoxib have shown less gastroentistinal toxicity [8].

Celecoxib successfully reduced the incidence and severity of postoperative pain following third molar surgery compared to ibuprofen and placebo [9].

Nonpharmacologic therapies like bromelain, baicalin, and escin, have been widely used for the treatment of inflammatory diseases and postsurgical conditions. They are efficient, safe, and have shown promising results in anti inflammation after third molar removal surgery [10].

Since the last decade, enzyme based drugs (anti-inflammatory) emerged, and have a great potential to be used. Serine protease, the largest proteolytic family has been reported for several therapeutic applications. Serratiopeptidase is a leading enzyme which has a very long history in medical use as an effective anti-inflammatory drug. Serratiopeptidase, which is an extracellular metalloprotease has an anti-inflammatory, antiedemic and fibrinolytic activity [11, 12]. The observation of it’s anti-inflammatory effects has led to its use in Japan for the first time [13]. It reduces inflammation and blocks the release of pain-inducing amines from inflamed tissues [11].

It has been used as a natural health product in Canada and as a dietary supplement in the US, the maximum dose of Serratiopeptidase is 60 mg/day. The recommended period is 1 week [14]. The usual adult dosage of Serratiopeptidase is 10 mg 3 times daily (range, 15 to 60 mg/day).

A recent meta-analysis based on five human clinical trials by Sivaramakrishnan and Sridharan suggested tremendous benefits of Serratiopeptidase after surgical removal of impacted molars [15]. Serratiopeptidase statistically reduced trismus after extraction in comparison with corticosteroids and ibuprofen [16]. Swelling was better with corticosteoroid in the first day and no significant results were noticed during the follow-up [17, 18]. However, Serratiopeptidase analgesic actions are the subject of debate and there is so far no conclusive statement on their efficacy.

To date, we can recognize the limitations imposed on assessing the potential clinical efficacy of Serratiopeptidase after third molar surgery. The challenge in analyzing current evidence stems from several factors, including the variety of dosing employed, and combinations with other drugs, which together create many combinations of factors that can result in differing clinical outcomes for patients [19], future clinical studies that will provide unequivocal answers to the role of Serratiopeptidase after dental surgeries are warranted.

The aim of this study was to assess the therapeutic effects of Serratiopeptidase after impacted third molar extraction.

## Methods

### Clinical trial design

The study was approved in the Institutional Review Board (IRB) at Jordan University for Science and Technology and Jordanian Food and Drug Administration. This randomized clinical trial was a single-centre, parallel-group, prospective, randomized, double-blind placebo-controlled study of Serratiopeptidase 10 mg, and placebo in patients undergoing third molar surgery who met the inclusion criteria. The study was performed in accordance with the International Conference on Harmonisation Good Clinical Practice and CONSORT guidelines. Strict inclusion criteria based on clinical and radiological examinations were applied in this study. The trial was prospectively registered with the clinicalTrials.gov, number NCT02493179. Patients with impacted third molars indicated for surgical extractions were recruited from consecutive adult patients attending Dental teaching clinics at a university hospital in northern Jordan from June 2015 till February 2016. The study included patients who agreed to participate in the study and fulfilled the following criteria:

1. Caucasian non-smoking patients aged between 18 and 50 years.
2. The presence of at least one asymptomatic mandibular third molar with class II position, type B impaction [20].
3. Absence of pericoronitis or signs of inflammation during the last 30 days.
4. No active diseases and good oral and general hygiene.

### Exclusion criteria

1. Other oral surgical procedures during the same session except the removal of supernumerary third molars.
2. Female subject who was pregnant or lactating.
3. Subject who has participated in any clinical research study within the previous 8 weeks.
4. Subjects on anti-coagulant drugs.
5. Participants Unwilling to continue the study and those with abnormality of wound healing process.
6. Subjects on any anti-inflammatory drugs for the last 7 days, other than Paracetamol.
7. Subject with a history of peptic ulcer

The required sample size was determined according to Snedecor and Cochran, 1980 equation. To detect a difference of 1.03 mm in swelling assuming a high coefficient of variation of more than 40% [21], after allowing for an estimated dropout rate of 10-20%, and admitting an alpha error of .05 and a beta of 0.2, a sample of 70 subjects was considered necessary per group.

Patients who met the inclusion criteria were assigned randomly into two groups through randomization.com: Group A received 10 mg of Serratiopeptidase tablets 3 times daily and Paracetamol 1000 mg 3 times daily after the surgery and group B, the control group, received Paracetamol (1000 mg 3 times daily) and placebo.

### Randomization

Randomization was practiced using the block computer random-number generator technique. The medication given to each participant in the study was blinded to the patient, surgeon, and clinical investigator responsible for follow-up and outcome examinations. In each patient, a lower impacted third molar was allocated in order to have one of the postoperative treatments. The allocation concealment was done through serially numbered envelopes, and the details of the sequence weren't known to any of the clinicians. Before every treatment, one of the authors, not involved in data recording and processing, performed the assignment of the sealed envelopes marked with the initials of the patient’s name, date of birth and suitable treatment methods. we stored black envelopes in a box containing a paper with the letter AA for group A or BB for group B. The envelopes were chosen for each patient after the extraction and transported to another room (without opening them); they were read only by one of the authors, who then distributed the treatment groups accordingly. The same operator performed all the procedures and was blinded to previously recorded data. If unblinding occurs, the surgeon must record the reason for unblinding, as well as the date and time of the event.

The researcher in charge of the clinical evaluation (H.K) was calibrated for intramural-examiner reproducibility in the examination procedure.

Four patient were examined at an interval of 48 hours. The interclass correlation coefficient was for the mean change in swelling and found to be above 90 percent

All patients signed written consent forms regarding the aims of the research before entering the study. The clinical investigator asked to report all Serious Adverse Events to the Competent Authorities (Jordan Food and Drug Administration) within 24 hours, and the Research Ethics Committee. Fatal or life-threatening Serious Adverse Events had to be reported within 7 days and all other Serious Adverse Events within 15 days. The CI was also informed all investigators concerned of relevant information about Serious Adverse Events that could adversely affect the safety of participants. All Serious Adverse Events was reported directly to safety monitoring committee (SMC) at Al-Hayat Pharmaceutical Company.

For each surgery, a mucoperiosteal flap was raised under LA using 3.6 ml of 2% lidocaine with 1:100,000 epinephrine. Surgical bur no. 4 was used to perform osteoctomy, while surgical bur no. 701 was used to section the tooth. The impacted tooth was then delivered using dental forceps. Surgical site was inspected and any sharp bone was filed to prevent discomfort. Copious irrigation followed by closure using 3.0 Poly galactin sutures. The surgeries were done in the morning from 8-11 am. All surgeries were done by the same surgeon (Z.T).

Trismus assessment was carried out by measuring changes in the maximal interincisal distance. Briefly, at predetermined time points post-surgery, the participant is asked to have their mouth wide open and then the interincisal distance is measured using Vernier calipers. The time points of measurement are 0, 1, 2, 4, and 5 days after surgery, where Day 0 records are facial measurements and interincisal distance before surgery.

The Laskin method [22] was applied to measure swelling based on distances at predetermined time points after surgery (0, 1, 2, 4, and 5 days). (H.K) measured trismus and swelling post operatively.

The distances are:

- The distance in millimeters from the bottom edge of the earlobe to the midpoint of the symphysis Hirota; horizontal distance to the symphysis (DHS).
- The distance in millimeters from the bottom edge of the earlobe to the external angle of the mouth; horizontal distance to the corner (DHC).
- The distance in millimeters from the palpebral outboard angle to the gonial angle; vertical distance (DV) [22].

Post-operative pain as measured by a verbal, numeric scale (0 to 10). where 0 is the least pain and 10 is the worst one. The pain measurements on days 0,1,2,4 and 5 were recorded through phone calls made by the clinical co-investigator, where Day 0 record is 6 h post surgery.

If postoperative infection or any side effect happens, the clinical investigator will report all Serious Adverse Events to the Competent Authorities (Jordan Food and Drug Administration) within 24 h, and the Research Ethics Committee. Fatal or life-threatening Serious Adverse Events must be reported within 7 days and all other Serious Adverse Events within 15 days. The CI will also inform all investigators concerned of relevant information about Serious Adverse Events that could adversely affect the safety of participants. All Serious Adverse Events was reported directly to safety monitoring committee (SMC) et al.-Hayat Pharmaceutical Company.

We instructed patients to use rescue medication only if necessary, because we do believe that alleviated and reduced symptoms can bias the difference in outcome between the placebo and the active treatment group. Furthermore, it is difficult to define the optimum dose when rescue medication is used. All patients were asked to report to the study investigator if they have intolerable pain to guide them to use rescue codeine 15 mg PO every 4 h. However, it must be borne in mind that, if the normal post-operative course occurs, then treatment is unlikely to cause symptoms, and thus the need for rescue medication will be minimized.

### Statistical analysis

Study data were summarized using descriptive statistics (e.g., mean, standard deviation, range, minimum and maximum). Age and gender were compared using t-test and chi-square test, respectively. For the comparison of data at different time points, multivariate analysis of variance (MANOVA) procedure was performed (Wilk’s Lambda) followed by t-test when the result was significant while age and gender were included as covariates. Statistical analysis was performed using Statistical Package for the Social Sciences version (IBM, SPSS version 20, 2011) and statistical significance was set at p < 0.05.

## Results

The study sample consisted initially of 140 patients; 76 were females (54.3%) and 64 were males (45.7%). Seventy patients were assigned to each of the two groups. The mean age of the study patients was 22.9 years (range 19–41 years). There was no difference between the two study groups in terms of age (P = 0.521) or sex distribution (P = 0.429) (Additional file 1: Table 1).

**Fig. 1:**
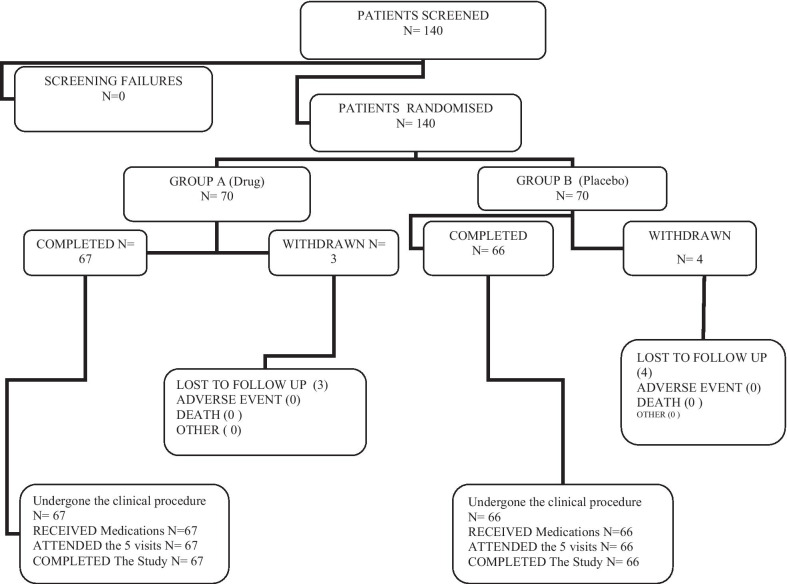
Participant flow

Patient participation and the reasons for withdrawal are summarized in Fig. 1.

Statistical significant difference of treatment group in comparison with placebo was noted on days 4 and 5 (P < 0.001). The mean mouth opening was 32.06 mm in the treatment group and 27.30 mm in placebo group on day 4, and 36.55 and 29.02, mm on day five, respectively (Table 1).

**Table 1:** Trismus measurements; mean ± SD values (millimetres)

Groups	Preoperative(Day 0)	Postoperative (Day 4)	(Day 5)
Test	45.84 ± 7.1	32.06 ± 7.7***	36.55 ± 7.8***
Control	44.84 ± 5.9	27.30 ± 7.3***	29.02 ± 7.5***
Effect size (d)	0.64	0.98	

Significance of treatment group in comparison with placebo on respective days

***p < 0.001

The means of the 3 distances; (DHS), (DHC) and (DV) measured, and the mean preoperative measurement (baseline) of all distances were calculated (Table 2).

**Table 2:** Mean of three distances (mm) measured over the swelling using Laskin method (mean ± SEM)

Groups	Preoperative (Day 0)	Postoperative (Day 4)	(Day 5)
DHC			
Test	108.41 ± 8.6	111.49 ± 8.1***	109.29 ± 8.2***
Control	109.98 ± 9.7	115.39 ± 9.9***	114.01 ± 9.8***
Effect size (d)	0.67	1.07	
DHS			
Test	147.14 ± 10.2	150.76 ± 9.1**	148.1 ± 9.1***
Control	146.01 ± 13.6	151.12 ± 14.2**	150.1 ± 14.1***
Effect size (d)	0.45	0.97	
DV			
Test	104.26 ± 13.4	106.17 ± 13.7***104.73 ± 13.5***	
Control	103.98 ± 9.9	107.42 ± 9.7***	106.19 ± 9.7***
Effect size (d)	0.69	0.98	

Significance of treatment group in comparison with placebo on respective days

**p < 0.01; ***p < 0.001

A statistical significant difference in DHC appeared on day four (111, 49 and 115.39 mm for treatment and placebo groups, respectively (P < 0.001). A significant difference was also shown on day 5 (P < 0.001) with the treatment group recording improvement in the distance (109.29 mm).

Day 4 showed a significant difference in DHS measurements between treatment and placebo groups (150.76 and 151.12 mm, respectively (P < 0.01). A further significant improvement in swelling was shown on day 5 (148.1 mm, P < 0.001).

Similar to both DHC and DHS, days 4 and 5 showed statistical significance in reduction of swelling (P < 0.001). Swelling was almost reduced to baseline by the treatment group on day 5 (104.73 mm) (Table 2)

$$\begin{aligned} & {\text{Measurement}}\,{\text{was}}\,{\text{re - calculate}}\,{\text{as}}\,{\text{following:}}\,\% {\text{recovery}} \\ & \quad = \left( {{\text{post - operative}}\,{\text{measurement}}{-}{\text{preoperative}}\,{\text{measurement}}} \right) \\ & \quad /{\text{preoperative}}\,{\text{measurement }} \times 100\% \\ \end{aligned}$$

**Fig. 2:**
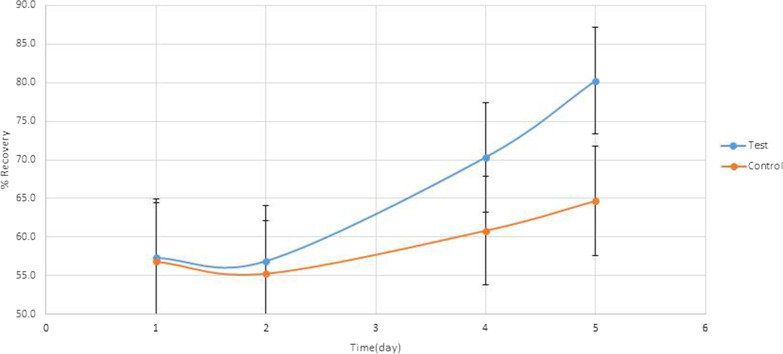
Trismus recovery

**Fig. 3:**
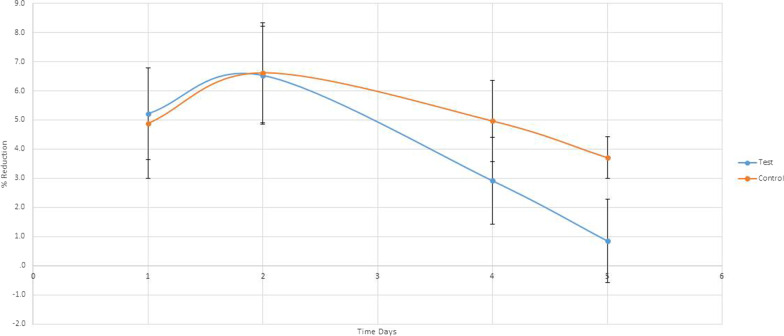
Percentage of DHC swelling change

**Fig. 4:**
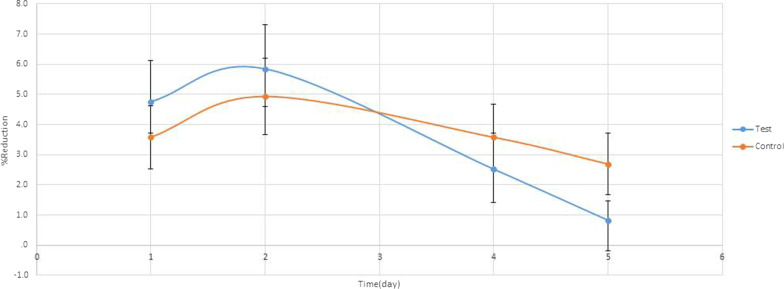
Percentage of DHS swelling change

**Fig. 5:**
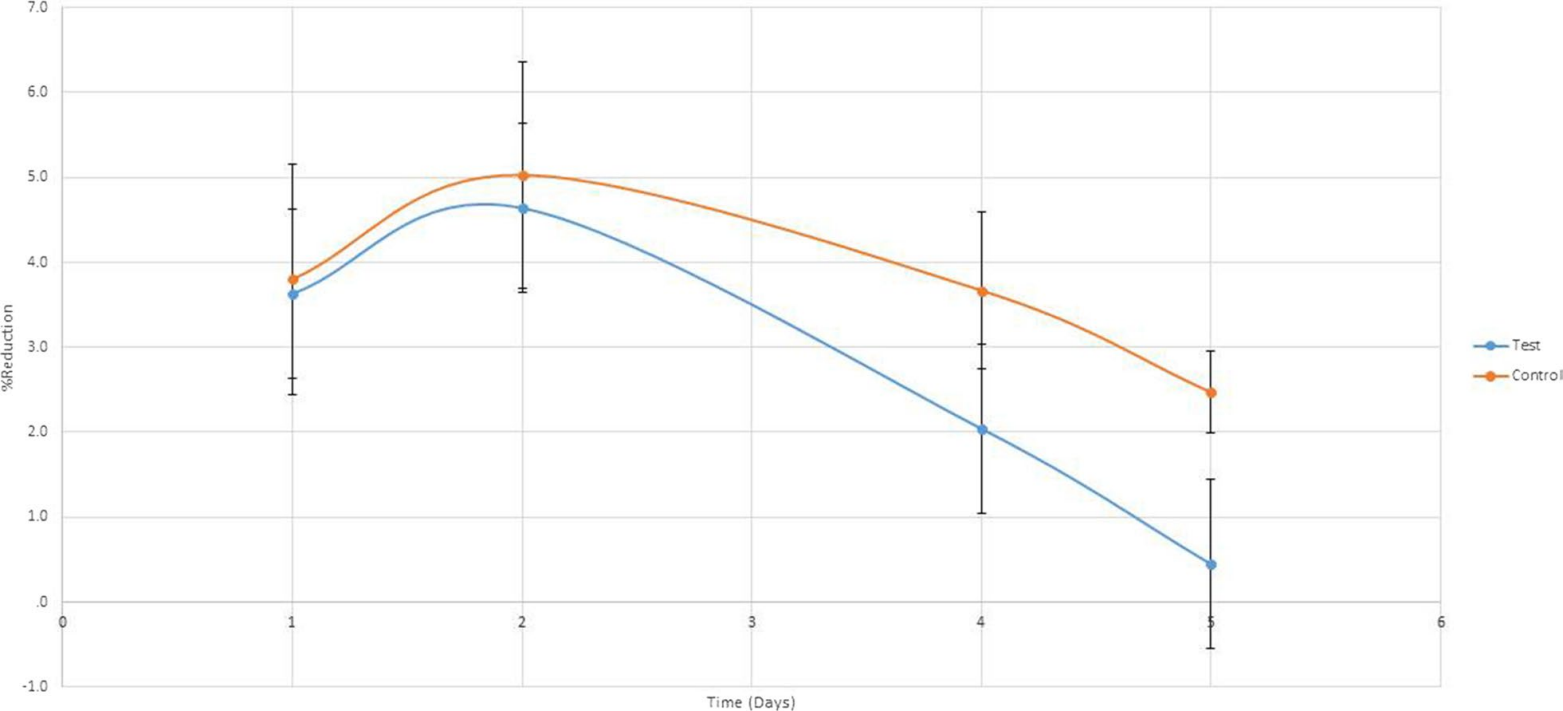
Percentage of DV swelling change

The data were statistically analyzed using MANOVA followed by t-test when the result was significant with the following findings:

As seen in Figs. 2, 3, 4 and 5, a significant difference in DV values with reference to the baseline on the fourth day, 2.04 ± 1.87 mm and 3.67 ± 2.54 mm, respectively (P < 0.001) and on the fifth day, 0.45 ± 0.97 mm and 2.47 ± 2.37 mm, respectively (P < 0.001). Also, a significant difference in the difference in DHC values with reference to the baseline on the fourth day, 2.91 ± 2.79 mm and 4.97 ± 2.96 mm, respectively (P < 0.0001) and on the fifth day, 0.86 ± 1.45 mm and 3.71 ± 2.86 mm, respectively (P < 0.001).

**Fig. 6:**
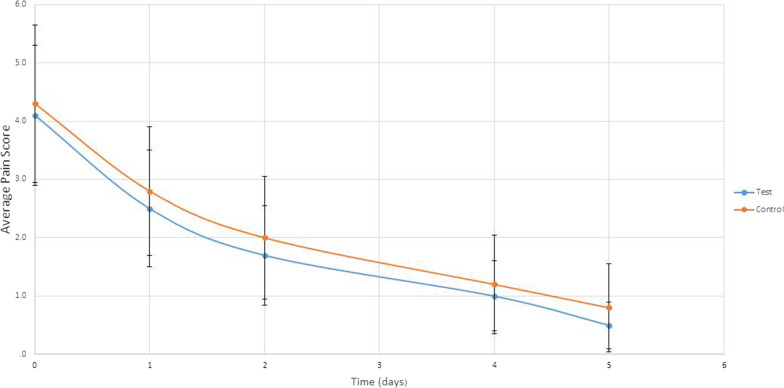
Average pain score

Pain showed no significant difference. As seen in Fig. 6.

Throughout the trial no side effects or rescue medications recorded.

## Discussion

It is now thought that the long exposure to NSAIDs frequently results in adverse effects on the liver and renal system. Therefore, researchers have been working on alternative therapies including the use of therapeutic enzymes. Serratiopeptidase, a proteolytic enzyme, has shown positive results in inflammatory disorders around teeth and dental implants [23–25].

To date, published studies assessing the efficacy of serratiopeptidase after third molar extraction are minor with inadequate methodological quality [16–18, 27].

In our study we followed Laskin method [22] to evaluate inflammation (trismus and edema). We carried assessments until the 5th day. Alkhateeb and Nusair [27] assessed swelling using cheek thickness only. Our results differed from those of Al Khateeb and Nusair [27] who didn’t find any changes in trismus, possibly because of the small number of patients examined. Furthermore, they carried the exam until the 7th day where trismus usually disappears. Also, in Al khateeb and Nusair study the dose for serropeptidase was only 5 mg which is the minimum dose to show effect.

The improved swelling noticed in our study could be due to the reported capacity of Serratiopeptidase to dissolve damaged and dead tissue produced in the healing processes [27, 28]. The enzyme was also reported to regulate recruitment of lymphocytes to the inflamed location. Moreover, it has been suggested that the enzyme may be responsible for the reduction of permeability induced by histamine and serotonin, assisting in breaking down exudates and proteins, and aiding in the absorption of decomposed products in lymphatics and blood [27].

In Chopra et al. [16] Serratiopeptidase showed analgesic and anti-inflammatory activity, but it was not significant. Possible reasons could be that in their study, 150 patients allocated in 5 groups so with this sample size there are possibilities that the power for detecting differences was not high enough. Also, all patients were given antibiotics. Intensity of inflammation usually declines at the end of the first postoperative week, so readings at the 7th day are not representative since tissue will go back to normal on its own. As far as pain, no significant results were detected, similar to Chappi et al. [18] who reported that methylprednisolone was superior in pain relief than serratioopeptidase.

In our results, the maximum inflammation intensities were observed on day two after surgery. The effect of Serratiopeptidase on postoperative inflammation appeared from the first postoperative day.

Compared to placebo, significant results were strongly shown on day 4 for improvement of trismus swelling. This is consistant with studies that have shown the efficacy of Serratiopeptidase in reduction of inflammation [16–18].

To further test the validity of our treatment effect, we compared our results with another study where patients took ibuprofen as historic controls, and found ibuprofen-treated subjects exhibited greater reduction in pain scores compared to our study group [29]. Possible explanation that Patients who receive substantial pain relief from ibuprofen have a more pronounced activation of the prostanoid biosynthetic pathway and regulation of the inflammatory pain phenotype differs from those patients who are taking Serratopeptidase and therefore may require other therapeutic intervention.

Previous research pinpointed some non-operative parameters such as sex, weight and body surface area of patients as potential risk factors and determinants of individual variation of extent of symptoms after third molar extraction [30]. In our study, no data concerning BMI was collected, but the sex was equally distributed between the 2 studied groups. The present study had a few limitations. Both subjects and controls were convenience samples recruited from the teaching dental hospital in northern Jordan with inherent selection bias, consequently limiting the applicability of our findings to the general population. It is noteworthy that in the inclusion criteria, we placed the upper limit of age as 50 while in recruitment only one patient was 42 while the rest where 35 and below. Controls were not age and gender matched. However, they did not have any significant demographic differences from the cases in the analysis.

## Conclusion

This clinical study provided evidence of the anti-inflammatory efficacy in the treatment of inflammation after third molar extraction. There was improvement in both trismus and swelling compared with the placebo. Future clinical studies might establish new indications for the use of serratiopeptidase in many clinical disorders.

## Supplementary Information

**Additional file 1: Table 1.** Demographics of the study patients

## Data Availability

The data collected is available and will be provided if asked for to the editors. Dr Zaid Tamimi ( the corresponding author) should be contacted if someone wants to request the data from this study.

## Abbreviations

DHS: The distance in millimeters from the bottom edge of the earlobe to the midpoint of the symphysis
Hirota: Horizontal distance to the symphysis.
DHC: The distance in millimeters from the bottom edge of the earlobe to the external angle of the mouth; horizontal distance to the corner.
DV: The distance in millimeters from the palpebral outboard angle to the gonial angle; vertical distance

## References

[1] Bui CH, Seldin EB, Dodson TB (2003). Types, frequencies, and risk factors for complications after third molar extraction. J Oral Maxillofac Surg.

[2] Sebastiani A, Todero S, Gabardo G (2014). Intraoperative accidents associated with surgical removal of third molars. Braz J Oral Sci..

[3] Ong CK, Lirk B, Tan CH (2007). An evidence-based update on nonsteroidal anti-inflammatory drugs. Clin Med Res.

[4] Juni P, Rutjes AW, Dieppe PA (2002). Are selective COX 2 inhibitors superior to traditional non steroidal anti-inflammatory drugs?. BMJ.

[5] Grossi GB, Maiorana C, Garramone RA (2007). Effect of submucosal injection of dexamethasone on postoperative discomfort after third molar surgery: a prospective study. J Oral Maxillofac Surg.

[6] Wolfe MM, Lichtenstein DR, Singh G (1999). Gastrointestinal toxicity of nonsteroidal antiinflammatory drugs. N Engl J Med.

[7] Tramer MR, Moore RA, Reynolds DJM (2000). Quantitative estimation of rare adverse events which follow a biological progression: A new model applied to chronic NSAID use. Pain.

[8] Silverstein FE, Faich G, Goldstein JL (2000). Gastrointestinal toxicity with celecoxib vs nonsteroidal anti-inflammatory drugs for osteoarthritis and rheumatoid arthritis: the CLASS study: a randomized controlled trial. Celecoxib Long-term Arthritis Safety Study JAMA.

[9] Isola G, Matarese M, Ramaglia L, Cicciù M, Matarese G (2019). Evaluation of the efficacy of celecoxib and ibuprofen on postoperative pain, swelling, and mouth opening after surgical removal of impacted third molars: a randomized, controlled clinical trial. Int J Oral Maxillofac Surg.

[10] Isola G, Matarese M, Ramaglia L, Iorio-Siciliano V, Cordasco G, Matarese G (2019). Efficacy of a drug composed of herbal extracts on postoperative discomfort after surgical removal of impacted mandibular third molar: a randomized, triple-blind, controlled clinical trial. Clin Oral Investig.

[11] Mazzone A, Catalani M, Costanzo M (1990). Evaluation of serratia-peptidase in acute or chronic inflammation of otorhinolaryngology pathology: a multicentre, double-blind, randomized trial versus placebo. J Int Med Res.

[12] Klein G, Kullich W (2000). Short-term treatment of painful osteoarthritis of the knee with oral enzymes. A randomized, double-blind study versus diclofenac. Chem Drug Invest.

[13] Yamasaki H, Tsuji K (1967). Siki Anti-inflammatory action of a protease, TSP, produced by serratia. Folia Pharmacol Japan.

[14] Health Canada. Drugs and Health Products. Natural Health Products Ingredients Database: Serrapeptase, 2014.

[15] Sivaramakrishnan G, Sridharan K (2018). Role of serratiopeptidase after surgical removal of impacted molar: a systematic review and meta-analysis. J Maxillofac Oral Surg.

[16] Chopra D, Rehan HS, Mehra P (2009). A randomized, double-blind, placebo-controlled study comparing the efficacy and safety of paracetamol, serratiopeptidase, ibuprofen and betamethasone using the dental impaction pain model. Int J Oral Maxillofac Surg.

[17] Murugesan K, Sreekumar K (2012). Sabapathy B Comparison of the roles of serratiopeptidase and dexamethasone in the control of inflammation and trismus following impacted third molar surgery. Indian J Dent Res.

[18] Chappi DM, Suresh KV, Patil MR (2015). Comparison of clinical efficacy of methylprednisolone and serratiopeptidase for reduction of postoperative sequelae after lower third molar surgery. J Clin Exp Dent.

[19] Bhagat S, Agarwal M, Roy V (2013). Serratiopeptidase: a systematic review of the existing evidence. Int J Surg.

[20] Pell GJ, Gregory BT (1933). Impacted mandibular third molars: classification and modified techniques for removal. Dent Dig.

[21] Shaikh M, Khatoon S, Rajput F, Yousif S, Shah A (2014). Impacted mandibular third molar surgery; the role of dexamethasone in postoperative swelling and trsimus. Profess Med J.

[22] Laskin DM (1987). Cirugía Bucal y Maxilofacial.

[23] Villafuerte-Nuñez AE, Téllez-Anguiano AC, Hernández-Díaz O et al. Facial edema evaluation using digital image processing. Discrete Dynamics in Nature and Society, 2013

[24] Seymour RA, Blair GS, Wyatt FA (1983). Post-operative dental pain and analgesic efficacy. Part I. Br J Oral Surg.

[25] Sannino G, Gigola P, Puttini M (2013). Combination therapy including serratiopeptidase improves outcomes of mechanicalantibiotic treatment of periimplantitis. Int J Immunopathol Pharmacol.

[26] Passariello C, Lucchese A, Pera F (2012). Clinical, microbiological and inflammatory evidence of the efficacy of combination therapy including serratiopeptidase in the treatment of periimplantitis. Eur J Inflamm.

[27] Al-Khateeb TH, Nusair Y (2008). Effect of the proteolytic enzyme serrapeptase on swelling, pain and trismus after surgical extraction of mandibular third molars. Int J Oral Maxillofac Surg.

[28] Jadav SP, Patel NH, Shah TG (2010). Comparison of anti-inflammatory activity of serratiopeptidase and diclofenac in albino rats. J Pharmacol Pharmacother.

[29] Aznar-Arasa L, Harutunian K, Figueiredo R, Valmaseda-Castellón E, Gay-Escoda C (2012). Effect of preoperative ibuprofen on pain and swelling after lower third molar removal: a randomized controlled trial. Int J Oral Maxillofac Surg.

[30] Akadiri OA, Okoje VN, Arotiba JT (2008). Identification of risk factors for short-term morbidity in third molar surgery. Odontostomatol Trop.

