# Supplementary material for: Efficacy of serratiopeptidase after impacted third molar surgery: a randomized controlled clinical trial
Source: BMC Oral Health. 2021 Mar 2;21:91. doi: 10.1186/s12903-021-01451-0 (PMC7927242; doi:10.1186/s12903-021-01451-0)
Supplement: Supplementary file 1 — Additional file 1: Table 1. Demographics of the study patients [file 12903_2021_1451_MOESM1_ESM.doc]

# Demographics of the Study Patients

|  |  | Group A(51244)  (N=) |  | Group B(150023)  (N=) |
| --- | --- | --- | --- | --- |
| Sex | Male | 34 |  | 30 |
|  | Female | 36 |  | 40 |
| Age (years) | n | 67 |  | 66 |
|  | Mean | 22.9 |  | 23.3 |
|  | Min | 19.0 |  | 18.00 |
|  | Max | 41.0 |  | 34.00 |
|  |  |  |  |  |
|  |  |  |  |  |

Sex and age were evaluated as confounding factors and were statistically analysed using MANOVA with the following findings:

| Effect | | Value | F | Hypothesis df | Error df | Sig. | Observed Power |
| --- | --- | --- | --- | --- | --- | --- | --- |
| Sex | Pillai's Trace | .232 | 1.659^b^ | 20.000 | 110.000 | .052 | .935 |
|  | Wilks' Lambda | .768 | 1.659^b^ | 20.000 | 110.000 | .052 | .935 |
|  | Hotelling's Trace | .302 | 1.659^b^ | 20.000 | 110.000 | .052 | .935 |
|  | Roy's Largest Root | .302 | 1.659^b^ | 20.000 | 110.000 | .052 | .935 |
| Age | Pillai's Trace | .226 | 1.604^b^ | 20.000 | 110.000 | .064 | .924 |
|  | Wilks' Lambda | .774 | 1.604^b^ | 20.000 | 110.000 | .064 | .924 |
|  | Hotelling's Trace | .292 | 1.604^b^ | 20.000 | 110.000 | .064 | .924 |
|  | Roy's Largest Root | .292 | 1.604^b^ | 20.000 | 110.000 | .064 | .924 |
| Treatment | Pillai's Trace | .716 | 13.852^b^ | 20.000 | 110.000 | .000 | 1.000 |
|  | Wilks' Lambda | .284 | 13.852^b^ | 20.000 | 110.000 | .000 | 1.000 |
|  | Hotelling's Trace | 2.519 | 13.852^b^ | 20.000 | 110.000 | .000 | 1.000 |
|  | Roy's Largest Root | 2.519 | 13.852^b^ | 20.000 | 110.000 | .000 | 1.000 |
